# MCT4/Lactate Promotes PD-L1 Glycosylation in Triple-Negative Breast Cancer Cells

**DOI:** 10.1155/2022/3659714

**Published:** 2022-09-26

**Authors:** Xianxian Duan, Yu Xie, Jing Yu, Xiao Hu, Zhanzhao Liu, Ning Li, Junfang Qin, Lan Lan, Mengci Yuan, Zhanyu Pan, Yue Wang

**Affiliations:** ^1^School of Medicine, Nankai University, Tianjin 300071, China; ^2^State Key Laboratory of Medicinal Chemical Biology & College of Pharmacy, Nankai University, Tianjin 300350, China; ^3^Institute of Disaster and Emergency Medicine, Tianjin University, Tianjin 300072, China; ^4^Department of Integrated Traditional & Western Medicine, Tianjin Medical University Cancer Institute and Hospital, Tianjin 300060, China; ^5^Tianjin Key Laboratory of Oral and Maxillofacial Function Reconstruction, Hospital of Stomatology, Nankai University, Tianjin 300041, China

## Abstract

Triple-negative breast cancer (TNBC) has the highest percentage of lymphocytic infiltration among breast cancer subtypes, and TNBC patients may benefit from anti-PD-1/PD-L1 immunotherapy. However, some cases whether the immune checkpoint blockade (ICB) shows low targeting efficiency have occurred and effective synergistic targets need to be found, which inspired our exploration of the co-expression analysis of MCT4 (SLC16A3) and PD-L1 (CD274) and their potential regulatory mechanisms. After bioinformatic analysis of the relationship between MCT4 and PD-L1, we validated their positive co-expression relationship in triple-negative breast cancer through multiple immunohistochemical staining (mIHC), CRISPR/Cas9, and lentiviral transduction for MCT4 knockout (sgMCT4/231 KO) or overexpression (pEGFP-N1-MCT4/231). We examined the effect of lactate treatment on PD-L1 expression in triple-negative breast cancer cells by qRT-PCR and Western blot. Combined with our results, we found that MCT4 positively regulated PD-L1 expression through discharging lactate and stabilized PD-L1 through promoting its glycosylation by the classic WNT pathway in MDA-MB-231 cells. More importantly, the high co-expression of MCT4 and PD-L1 appears to predict more effective targets for treating TNBC, which would improve immune checkpoint therapy for TNBC.

## 1. Introduction

TNBC, defined as no estrogen receptor (ER), no progesterone receptor (PR), and no human epidermal growth factor receptor-2 (HER2), accounts for 15–20% of all breast cancer (BC), but is much more problematic than other molecular subtypes of breast tumors and has a poor prognosis [1–3].

Immune checkpoint blockade (ICB) has now revolutionized the cancer therapy, notably melanoma, lymphoma, renal cell carcinoma, non-small cell lung cancer, and TNBC [4]. Immune checkpoint protein programmed cell death 1 (PD-1) is mainly expressed in T cells [5], which binds programmed cell death 1 ligand 1 (PD-L1) on antigen-presenting cell or cancer cell. Studies have shown that the glycosylated modifications keep PD-L1 stable on cell membranes [6], increasing the chance that PD-1 recognizes and binds to PD-L1 and promoting immune escape of cancer cells [4, 7]. A study in 650 BC cases presents that high expression of PD-L1 correlates with diminished overall survival and poor prognosis [8, 9]. The upregulation of PD-L1 is regarded as the amplification of PD-L1 gene (CD274) at 9p24.1, which has been confirmed in TNBC cells (TBcs) [10]. The high expression of PD-L1 in several cancers including TNBC pioneers the clinical use of PD-L1 or PD-1 inhibitors, such as nivolumab [11–13].

Monocarboxylate transporter protein 4 (MCT4) primarily mediates the transmembrane transport of proton-coupled monocarboxylic acid, such as lactate, and the high expression of MCT4 promotes cancer progression [14, 15]. TBcs exhibit a higher glycolysis rate due to high-glucose uptake rate, overexpression of glycolytic enzymes, high oxygen consumption rate (OCR), and high extracellular acidification rate (ECAR) [16–18]. Accordingly, MCT4 is highly expressed in triple-negative breast cancers to excrete metabolic waste generated by the glycolytic pathway [18, 19]. Therefore, MCT4 is considered as a potential oncogene and its structure is being studied for new strategies that block it [20].

Most breast cancer cells exhibit a distinct pro-proliferative and invasive metabolic profile, which is referred as metabolic reprogramming [21], and altered metabolism has profound effects on the tumor microenvironment [22]. For example, a high rate of glycolysis increases the production and accumulation of lactate regardless of oxygen concentration [23, 24]. Tumor-produced lactate is internalized by cytotoxic T cells (CTLs) and inhibits their proliferative and anticancer functions by suppressing the activation of p38 and JNK/c-Jun, which are required for IFN-*γ* production [25]. Lactate also polarizes macrophages to a M2 protumor phenotype and exerts immunosuppressive effects by activating G protein-coupled receptors 132. In addition, lactate inhibits the differentiation of monocytes to dendritic cells, thereby preventing dendritic cells (DCs) from exerting anticancer effects. Thus, elevated lactate levels are not only a byproduct of breast cancer glycolysis but also attenuate the anticancer immune response in a concentration-dependent manner and play a key role in the regulation of the tumor microenvironment [26]. It was found that MCT4-related genes are involved not only in metabolism but also in immune-related biological pathways, such as myeloid leukocyte activation, the acquired immune system, and catabolic processes. Expression of MCT4 was significantly correlated with breast cancer immune infiltration. The TIMER database showed that MCT4 expression was associated with dendritic cell infiltration in all breast cancer patients (correlation = 0.351) and the basal-like subtype (correlation = 0.316), and macrophage and B-cell infiltration in the HER2^+^ subtype correlated with MCT4 expression (correlation coefficients of 0.328 and −0.385, respectively) [26]. These findings suggest a modest correlation between MCT4 expression, macrophages, B cells, and dendritic cells. A recent study reported that downregulation of MCT4 promoted cytotoxicity of NK cells in breast cancer [15], and another study showed that the immunosuppressive effect of MCT4 might be caused by suppression of macrophage maturation or interference of T-cell metabolism [27]. Notably, scientists have identified MCT4, PD-L1, CD163, and FOXP3 as important markers for the prognosis of TNBC [28], which not only redefines markers for molecular subtypes of breast cancer but facilitates progress for dual-targeted therapy research in triple-negative breast cancer.

It is now recognized that acidic tumor microenvironment (TME) and extracellular lactate can promote tumor metastasis and help cancer cell escape immunologic surveillance [29–34], which implies a connection between glycolytic metabolic molecules and immune-related molecules. The expression of PD-L1 is reported to be sensitive to lactate [35]. On this basis, we intend to study the correlation of MCT4 with PD-L1 and explicate the role of lactate in the expression and stabilization of PD-L1, which may illustrate novel composite therapeutic targets for TNBC.

## 2. Materials and Methods

### 2.1. Gene Enrichment

*CD274* (PD-L1) and *SLC16A3* (MCT4) were performed using the Metascape online database (https://www.metascape.org/) based on the dataset retrieved from the STRING database (https://string-db.org/). We utilized UALCN-TCGA (The Cancer Genome Atlas) online database (https://ualcan.path.uab.edu/cgi-bin/ualcan-res.pl) to search the expression level of MCT4 and PD-L1 in BC and TNBC.

### 2.2. Cell Culture

The human TNBC cell lines (TBcls) MDA-MB-231, MDA-MB-468, and BT-549 were cultured in RPMI 1640 (BI) with 10% fetal bovine serum and then were treated with lactate in high-glucose DMEM (Dulbecco's modified Eagle's medium) following 12 hours (h) of glucose starvation, or with MCT4 inhibitor 7-aminocarboxycoumarins (7ACC1) (MCE, Shanghai, China). MDA-MB-231 was treated with tunicamycin (TM) (MCE, Shanghai, China), a protein glycosylation inhibitor that inhibits the formation of N-glycosidic linkages in glycoprotein synthesis, with 0.4 *μ*g/mL for 24 h, so was done with XAV939 (MCE, Shanghai, China), a WNT pathway inhibitor, with 10 *μ*M for 10 h.

### 2.3. Multiple Immunohistochemical Staining (mIHC)

After being fixed with 4% polyoxymethylene and dehydrated, the tissues were embedded with paraffin. Next, paraffin-embedded tissues were cut into 5 *μ*m slices and collected on adhesion slides. Following deparaffinization and rehydration, where slides were bathed in 100% xylene for 10 min, 100% xylene for 10 min, 100% ethanol for 5 min, 100% ethanol for 5 min, 95% ethanol for 10 min, 85% ethanol for 10 min, and finally tap water, the slides were soaked in antigen repair buffer using a high-temperature method. Then, 3% H_2_O_2_ solution was used for the removal of endogenous peroxidase. mIHC staining was conducted using anti-MCT4 (G-9, IgG2b*κ*) (Santa Cruz, sc-376139, 1 : 100), PD-L1 (CST, #13684, 1 : 400), and EpCAM (CST, #46403,1 : 400). After incubation with secondary antibody and TSA fluorescent dye, photographing, and then re-staining, mIHC images can be obtained. We trusted WiSee Biotechnology Company to perform mIHC staining and assay, which provided simultaneous detection and quantization of PD-L1, MCT4, and EpCAM on TNBC patient (TBp) tissue sections.

### 2.4. Western Blots (WBs)

The extraction of membranous and cytoplasmic protein was performed according to the kit (KeyGen Biotech, Jiangsu, China) instructions. Membranes were incubated with the primary antibodies anti-MCT4 (G-9, IgG2b*κ*)/anti-MCT4 (G-7, IgG3*κ*) (Santa Cruz Biotechnology, USA, 1 : 1500), anti-PD-L1 (Proteintech Group, Chicago, USA, 1 : 1000), anti-GAPDH (Zen Bioscience, 1 : 1000), and rabbit polyclonal anti-Caveolin-1 antibody (1 : 200, cat. no. 340158, Zen-Bio, Chengdu, China) overnight at 4°C. Membranes were scanned by Tanon 5200 apparatus (Tanon, Shanghai, China) with ECL luminescence kit (Millipore, Massachusetts, USA).

### 2.5. Extracellular Lactate and pH Measure

The lactate concentration of the supernatant was detected by Human Lactate ELISA Kit (J&L, Shanghai, China). The pH value of the supernatant was measured by PP-15-P11 Sartorius pH Meters (Germany). The pH detection range was −2.000 to 20.000. The pH default resolution and confidence were ±0.001. All samples were assayed three times.

### 2.6. Quantitative Real-Time PCR (qRT-PCR)

Total RNA was extracted using lysis reagent TRIzol Kit (Vazyme, Nanjing, China), and cDNA was obtained by HiFair® II 1st Strand cDNA Synthesis Kit (Yeasen, Shanghai, China). qRT-PCR was carried out using UltraSYBR Mixture (Cwbio, Beijing, China), and data were analyzed by the ΔΔCt method. The primers are listed in Table 1.

### 2.7. Flow Cytometry (FAC)

The treated cells were trypsinized and stained with FITC conjugated anti-mouse PD-L1 (BioLegend, San Diego, CA, USA) or with mouse IgG-FITC (BD Biosciences, San Jose, CA, USA) and analyzed by a flow cytometer, and data obtained were presented with FlowJo software.

### 2.8. CRISPR-Cas9-Mediated Gene Editing

MCT4 small-guide RNA (sgRNA) oligo-sequences (https://crispr.mit.edu:8079/) were acquired (Table 2). The recombinant plasmid of lenti-CRISPR V2 was inserted with MCT4 sgRNA, and it was transfected into MDA-MB-231 cells via Lipofectamine® 2000 reagent (Invitrogen, Carlsbad, California, USA) for generating MCT4 knockout (KO) in MDA-MB-231 cells, which was selected by puromycin (3 *μ*g/mL).

### 2.9. Establishing the Stable MDA-MB-231 Cells Expressing MCT4

Full-length MCT4 was cloned into pEGFP-N1 (Shanghai GeneChem, Shanghai, China). The plasmid pEGFP-N1-MCT4-wild type (wt) was transfected into HEK-293T cell lines via Lipofectamine® 2000 reagent. The virus supernatant was subsequently transfected to MDA-MB-231 cells for 48 h to overexpress MCT4, and the infected cells were selected with Geneticin (20 *μ*g/mL).

### 2.10. Cell Viability

Cells were seeded into 96-well plate (1 *∗* 10^5^ cells per well) and incubated overnight at 37°C. The cells were incubated with lactate, 7ACC1, TM, or XAV939 and then analyzed by CCK-8 assay (ab228554, Abcam).

### 2.11. Mice and In Vivo Treatments

Five-week-old BALB/C female mice were injected subcutaneously with 1 × 10^5^ 4T1 breast cancer cells into the mammary fat pad of mice, which were randomly divided into four groups. When tumors reached a mean diameter of 5 mm, 7ACC1 (0.3 mg/kg, intraperitoneally, daily) or/and PD-L1 inhibitor fraxinellone (30 mg/kg, oral gavage, every three days) were administrated for 14 days. Tumor sizes were tracked with an electronic calliper and determined using the formula: length × width^2^ × 0.52. Each procedure was approved by Institute Research Ethics Committee at Nankai University according to National Animal Care regulations.

### 2.12. Statistical Analysis

One-way ANOVA and Student's *t*-test are performed using SPSS 13.0 Statistical Software (SPSS Inc., Chicago, IL, USA) and are presented as mean ± s. d. from triplicated independent experiments. A significant difference was considered when the *P* value was <0.05.

## 3. Results

### 3.1. MCT4 and PD-L1 Were Functionally Linked and Highly Expressed in TNBC Patient Samples

First, we chose the STRING online database (https://string-db.org/) to retrieve genes related to MCT4 and PD-L1, respectively. A comprehensive analysis of the two gene sets was then performed using the Metascape online database (https://www.metascape.org/), which showed a dense functional overlap between the two gene sets (Figure S1A). The heat map of the enrichment analysis (Figure S1B) shows that the two gene sets are co-enriched in carbon metabolism pathway in tumor. A total gene set composed of the two gene sets was enriched in PD-1 signaling pathway (Figure S1C). All these results indicate that MCT4 and PD-L1 are functionally related, providing a strong theoretical basis for our subsequent basic experiments.

**Figure 1:**
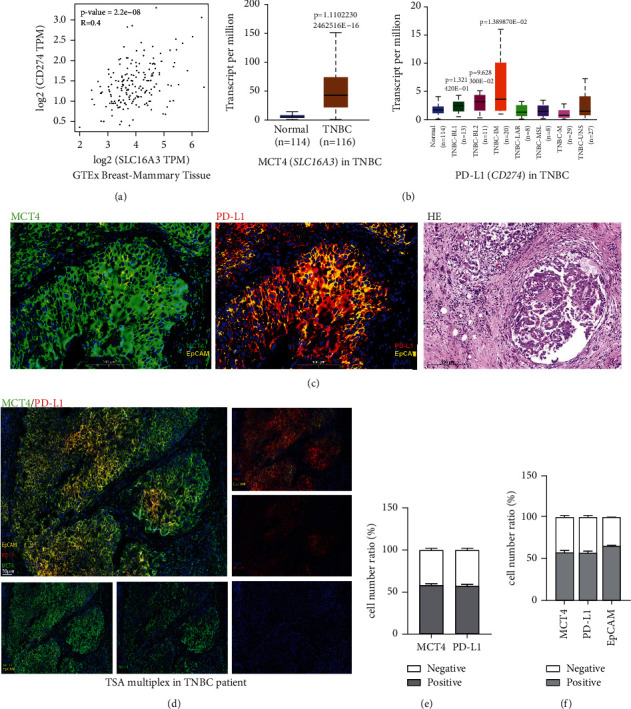
MCT4 and PD-L1 were functionally linked and highly expressed in TNBC patient samples. (a) Relativity of MCT4 and PD-L1 in BC was exhibited via the GTEx database. (b) MCT4 and PD-L1 expression in BC based on major subtypes (with TNBC types) was presented through the TCGA database. (c) The distribution of MCT4 and PD-L1 in the tissue of TBps was imaged by mIHC and HE staining. (d) The co-expression of MCT4 and PD-L1 was presented in mIHC image. (e, f) The fluorescence quantitation of MCT4, PD-L1, and EpCAM in TBp tissue.

In this context, we analyzed the correlation between MCT4 and PD-L1 in BC utilizing GEPIA and found that their expression was positively correlated (*R* = 0.4, Figure 1(a)). Meanwhile, we found that MCT4 and PD-L1 were co-overexpressed in TNBC depending on the TCGA database (*P* < 0.05, Figure 1(b)).

Further, we performed mIHC analysis and HE staining in tumor tissues from TBps (*n* = 3) and analyzed the correlation between MCT4 and PD-L1 expressions (*n* = 3) (Supplementary Material 1). Stromal and infiltrating lymphocytes of TNBC tumor were outlined on full-face HE sections, and MCT4 and PD-L1 were co-located in TBp tissues (Figure 1(c)). Image analysis presented that the co-expression rate of MCT4 and PD-L1 was over 50% in TNBC samples (Figures 1(d)–1(f)). The percentage of MCT4^+^EpCAM^+^, PD-L1^+^EpCAM^+^, and MCT4^+^PD-L1^+^EpCAM^+^ cells in tumor tissues was higher than 50% as shown in typical case 2 (Supplementary Material 2 and Figure S2), and EpCAM was used as a marker for poor prognosis of TNBC. The positive correlation between MCT4 and PD-L1 inspired us to consider whether MCT4 could regulate the expression of PD-L1.

### 3.2. MCT4 Promotes Lactate Efflux to Maintain Acidic TME in TNBC Cell Lines

**Figure 2:**
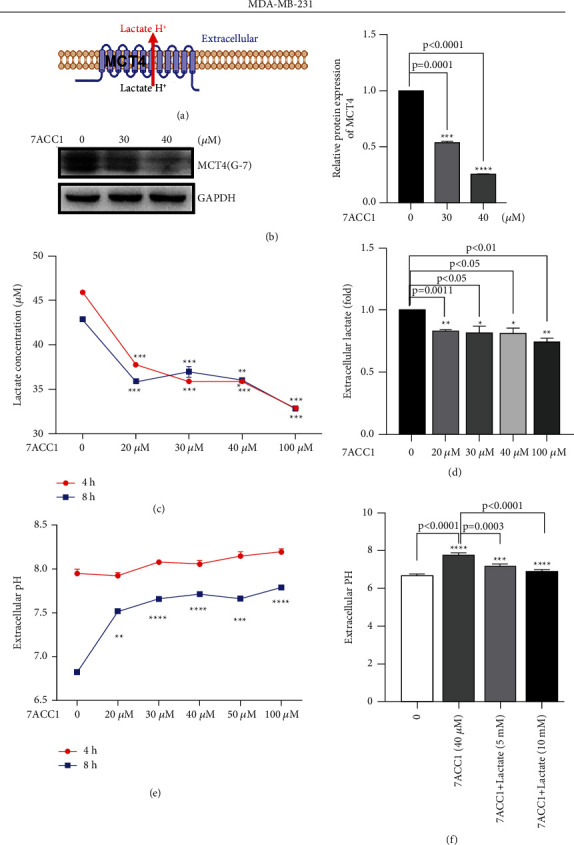
MCT4 promoted lactate efflux and sustained an acidic TME in MDA-MB-231 cells. (a) MCT4, a high-affinity transporter capable of exporting lactate in high-lactate microenvironments. (b) WB showed that protein levels of MCT4 and GAPDH in cells were treated with 7ACC1 (30 *μ*M and 40 *μ*M). (c, d) ELISA showed that fold and concentration of lactate in cells were treated with 7ACC1 (20 *μ*M–100 *μ*M) for 4 h and 8 h. (e) pH meter showed that pH value in cells was treated with 7ACC1 (20 *μ*M–100 *μ*M) for 4 h and 8 h. (f) pH meter showed that pH value in cells was treated with 7ACC1 (40 *μ*M) with/without lactate (5 mM) for 8 h.

In the beginning, we examined the expression of MCT4 in three human TBcls MDA-MB-231, MDA-MB-468, and BT-549 by WB (Figure S3). We then treated MDA-MB-231 cells with the MCT4 inhibitor 7ACC1 (30 *μ*M and 40 *μ*M) [36] and observed its inhibition in MCT4 by WB (*P* < 0.0001, Figures 2(a) and 2(b)). The extracellular lactate concentration decreased (*P* < 0.05, Figures 2(c) and 2(d)) along with the addition of 7ACC1 (20 *μ*M, 30 *μ*M, 40 *μ*M, and 100 *μ*M) for 4 h and 8 h [15]. Correspondingly, we observed that the acidic environment in the supernatant of cancer cells was reversed and pH value increased at 8 h after 7ACC1 inhibition (*P* < 0.01, Figure 2(e)), while the addition of lactate could rescue certain acidity, which indicated that lactate played a vital role in the formation of the whole acidic environment for cancer (*P* < 0.001, Figure 2(f)).

**Figure 3:**
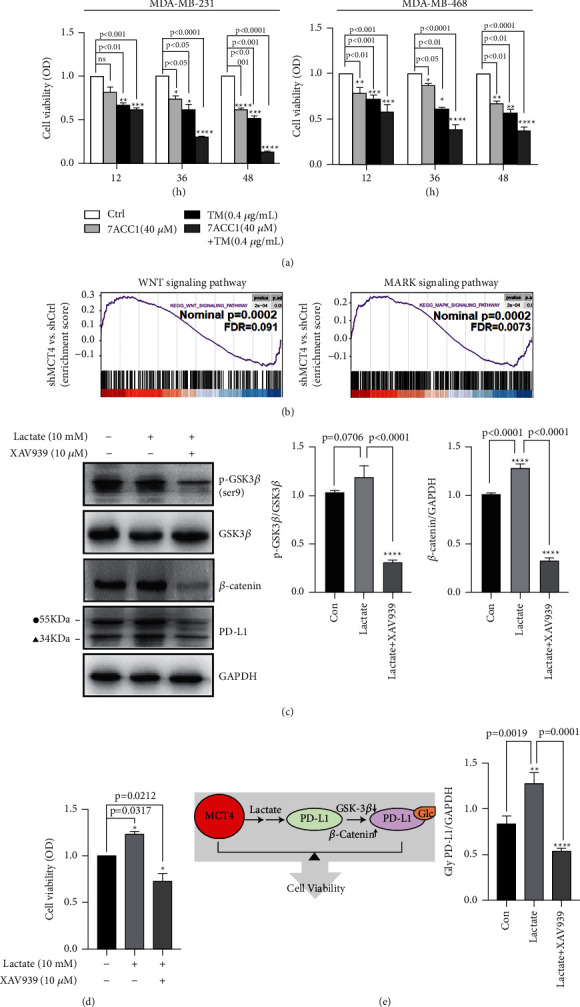
MCT4/lactate promoted PD-L1 N-glycosylation via activating the WNT pathway in MDA-MB-231 cells. (a) CCK-8 showed that the cell viability of cells was treated with 7ACC1 (40 *μ*M) with/without TM (4 *μ*g/mL) for 12 h, 36 h, and 48 h in 231 cells and 468 cells. (b) GSEA plot of the WNT pathways (FDR = 0.091) and MAPK pathways (FDR = 0.0073) based on the shMCT4 versus shControl translation efficiency profiles. (c) WB showed that protein levels of p-GSK3*β* (ser9) (a deactivation loci), GSK3*β*, *β*-catenin, and PD-L1 and GAPDH in 231 cells were treated by lactate (10 mM) with/without XAV939 (10 *μ*M) for 10 h. 55 KDa form (●) represents glycosylation of PD-L1, and 34 KDa form (▲) represents non-glycosylated PD-L1. (d) CCK-8 showed that the cell viability of cells was treated with lactate (10 mM) with/without XAV939 (10 *μ*M) for 10 h. (e) Schematic of MCT4/lactate in regulated PD-L1 expression in TBcs.

This suggested that high expression of MCT4 in cancer cells can lead to efflux of lactic acid and the proliferation of cancer cells in acidic environment (*P* < 0.01, Figure3(a)). It was shown that specific blockade of MCT4 significantly altered the acidic environmentwhere cancer cell depends so that affects the expression and functional properties of other molecules.

### 3.3. PD-L1 Was Sensitive to the Addition of Lactate in TNBC Cell Lines

**Figure 4:**
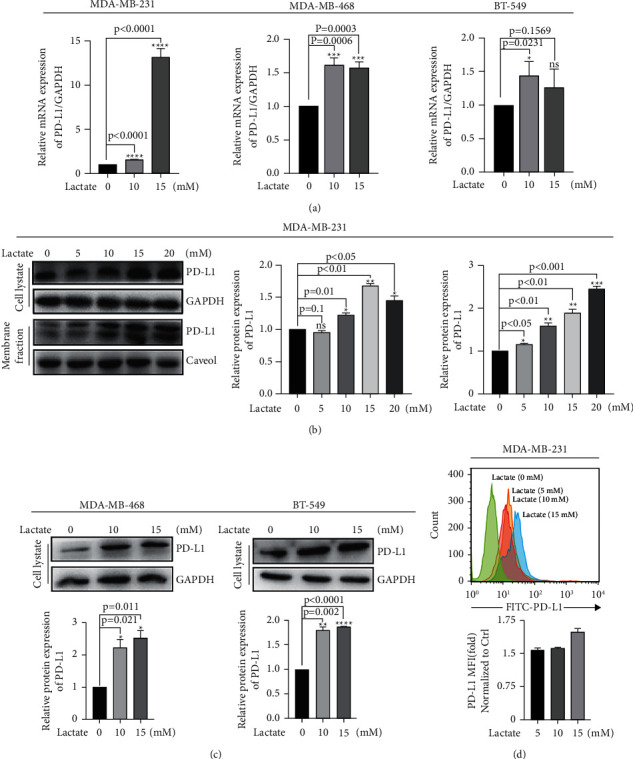
PD-L1 was expressed and sensitive to the addition of lactate in three TNBC cell lines. (a) qRT-PCR showed that mRNA level of PD-L1 in cells was treated with lactate (0, 10 mM, 15 mM). (b) WB showed that whole protein levels and membrane fraction levels of PD-L1 and GAPDH in 231 cells were treated with lactate (0, 5 mM, 10 mM, 15 mM, and 20 mM). (c) WB showed that protein levels of PD-L1 and GAPDH in 468 and 549 cells were treated with lactate (0, 10 mM, 15 mM), respectively. (d) The increased PD-L1 expression on cell membrane after lactate addition was presented by FACS analysis.

To determine whether PD-L1 expression is regulated by MCT4/lactate, we validated the sensitivity of PD-L1 expression to lactate. PD-L1 was expressed in all three TBcls MDA-MB-231, MDA-MB-468, and BT-549 (Figure S4). Then, we added lactate of gradient concentrations (5 mM, 10 mM, 15 mM, and 20 mM) to stimulate the cells and observed increased PD-L1 mRNA expression (*P* < 0.05, Figure 4(a)); accordingly, membrane PD-L1 protein and total PD-L1 protein increased in MDA-MB-231 (*P* < 0.05, Figure 4(b)), MDA-MB-468, and BT-549 cells (*P* < 0.05, Figure 4(c)). Simultaneously, a representative result of FAC showed the same upward trend of PD-L1 in the cell membrane of MDA-MB-231 cells after lactate treatment (*P* < 0.05, Figure 4(d)).

**Figure 5:**
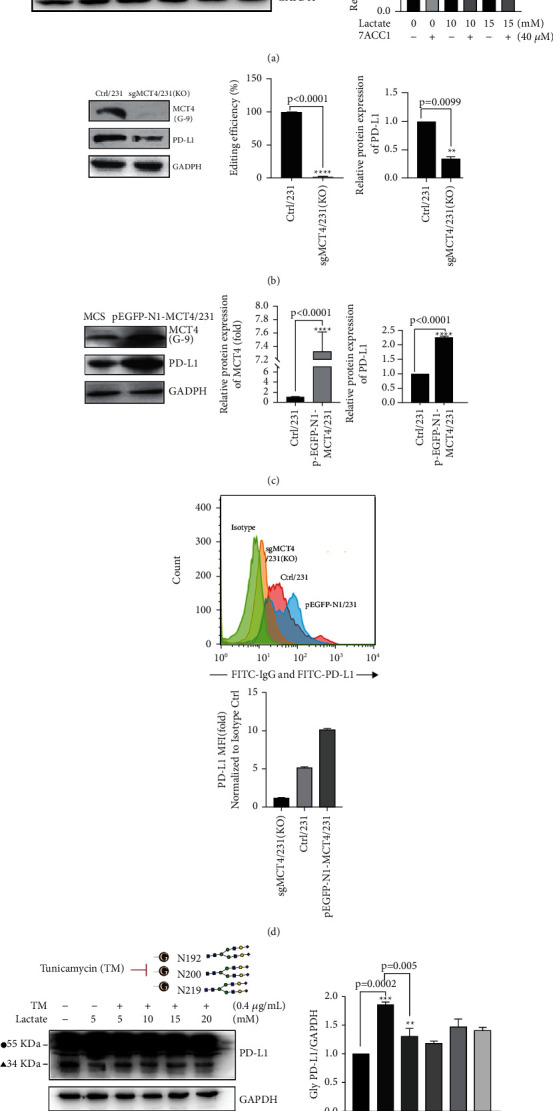
Regulation of PD-L1 expression or PD-L1 glycosylation by MCT4 is mediated through lactate. (a) WB showed that protein levels of MCT4, PD-L1, and GAPDH in 231 cells were treated by lactate (0, 10 mM, 15 mM) with/without 7ACC1 (40 *μ*M). (b) CRISPR/Cas9 was performed to knockout (KO) MCT4, and qRT-PCR was used to detect KO effect. WB showed that protein levels of MCT4, PD-L1, and GAPDH in 231 cells as MCT4 were KO. (c) qRT-PCR verified the construction of pEGFP-N1-MCT4 lentiviral vector and stable MCT4 overexpression cell line pEGFP-N1-MCT4/231. WB showed protein levels of MCT4, PD-L1, and GAPDH in pEGFP-N1-MCT4/231 cells. (d) FAC result shows the decreased or increased PD-L1 expression in sgMCT4/231 (KO) or pEGFP-N1-MCT4/231 cell lines. (e) TM, a N-linked glycosylation inhibitor. 55 KDa form represents glycosylation of PD-L1, solid sphere labeled (●), and 34 KDa form represents non-glycosylated PD-L1, filled triangle labeled (▲). 12% SDS-PAGE separated PD-L1 protein from 55 kDa to 34 kDa following lactate (0, 5 mM, 10 mM, 15 mM, and 20 mM) and TM (4 *μ*g/mL) treatment.

We further observed that the upward expression trend of PD-L1 disappeared after 7ACC1 (40 *μ*M, 8 h) treatment in MDA-MB-231 cells (*P* < 0.05, Figure 5(a)) despite lactate stimulation. Thus, this suggests that PD-L1 is sensitive to the addition of lactate only in the presence of MCT4 of normal or high expression. If MCT4 was blocked by 7ACC1 and the acidic environment was altered (pH > 7.5, Figure 2(e)), PD-L1 was no longer sensitive to the addition of lactate. It probably inferred that MCT4 dominantly upregulated PD-L1 expression by contributing to the acidic TME.

### 3.4. MDA-MB-231 Cell Lines of KO or Overexpressing MCT4 Were Established to Validate the Effect of MCT4/Lactate on PD-L1

To clearly demonstrate the hypothesis that MCT4 dominates the upregulation of PD-L1 expression, we specifically knocked down MCT4 in MDA-MB-231 cells using CRISPR/Cas9 and screened the stable MCT4-KO cell line sgMCT4/231 (KO) with interference efficiency of 98.5% ± 0.6%. Impressively, WB results showed that PD-L1 protein expression was significantly downregulated in sgMCT4/231 (KO) cells compared with controls (*P* < 0.001, Figure 5(b)). On the other hand, infectious lentivirus particles for MCT4 overexpression were constructed through transfecting 293T cells, and then, MDA-MB-231 cells were infected with the viral supernatant to obtain a stable MCT4 overexpression cell line pEGFP-N1-MCT4/231 with an expression level of 7.5 ± 1.5-fold. Further, WB results showed that PD-L1 protein expression was significantly upregulated in pEGFP-N1-MCT4/231 cells compared with controls (*P* < 0.0001, Figure 5(c)). Utilizing the cell lines of sgMCT4/231 (KO) or pEGFP-N1-MCT4/231, the same result was presented by FAC to prove the down- or upregulation of PD-L1 on the membrane following MCT4 inhibition or overexpression (*P* < 0.05, Figure 5(d)), which provides a plausible argument that MCT4 regulates PD-L1 expression as a upstream regulator.

### 3.5. MCT4/Lactate Promoted the Level of PD-L1 N-Glycosylation in MDA-MB-231 Cells

MCT4/lactate significantly upregulated the protein expression of PD-L1, but whether the acidic environment caused by the high expression of MCT4 would promote the protein glycosylation of PD-L1 so that make it more stable. Based on this hypothesis, we employed N-linked glycosylation inhibitor TM (0.4 *μ*g/mL) to treat MDA-MB-231 cells for 24 h and performed WB detection. Then, we found that with the TM addition, glycosylated PD-L1 protein decreased despite increased lactate concentration (*P* < 0.001, Figure 5(e)); correspondingly cell growth was suppressed with the decrease in glycosylated PD-L1 protein following the addition of TM in MDA-MB-231 and MDA-MB-468 cell lines (*P* < 0.001, Figure 3(a)).

### 3.6. MCT4/Lactate Promoted PD-L1 N-Glycosylation via Activating the WNT Pathway in MDA-MB-231 Cells

We further explore the molecular mechanisms mediating the regulation of PD-L1 by MCT4/lactate. GSEA was used to investigate the signaling pathways affected by MCT4 knockout and revealed that the related downregulation pathways were involved in the WNT and MAPK signaling pathways (FDR *q* < 0.25, Figure 3(b)). Based on the study that the WNT signaling pathway could modulate PD-L1 expression, we detected the protein expression of PD-L1 following treatment with the WNT pathway-specific inhibitor XAV939 under acidic conditions [37–39]. We found that lactate was able to activate the classical WNT signaling pathway through inactivating GSK3*β* and activating *β*-catenin, while the simultaneous addition of XAV939 (10 *μ*M) for the inhibition of WNT pathway decreased the levels of p-GSK3*β* (ser9) and *β*-catenin, and the protein expression of PD-L1 synchronously decreased (*P* < 0.001 vs. lactate, Figure 3(c)). Besides, cell viability tests showed that the inhibition of WNT pathway interfered with cell growth, which was consistent with the effect of 7ACC1 and TM (*P* < 0.05 in 36 h and 48 h, Figure 3(a), and *P* < 0.01, Figure 3(d)). Overall, the use of these three drugs showed simultaneous inhibition of tumor cells, and it suggests that the upregulation of WNT/PD-L1 in acidic TME induced by MCT4 jointly constitutes the malignant phenotype of TNBC (Figure 3(e)).

### 3.7. Inhibition of MCT4 and PD-L1 Hindered the Tumor Formation In Vivo

**Figure 6:**
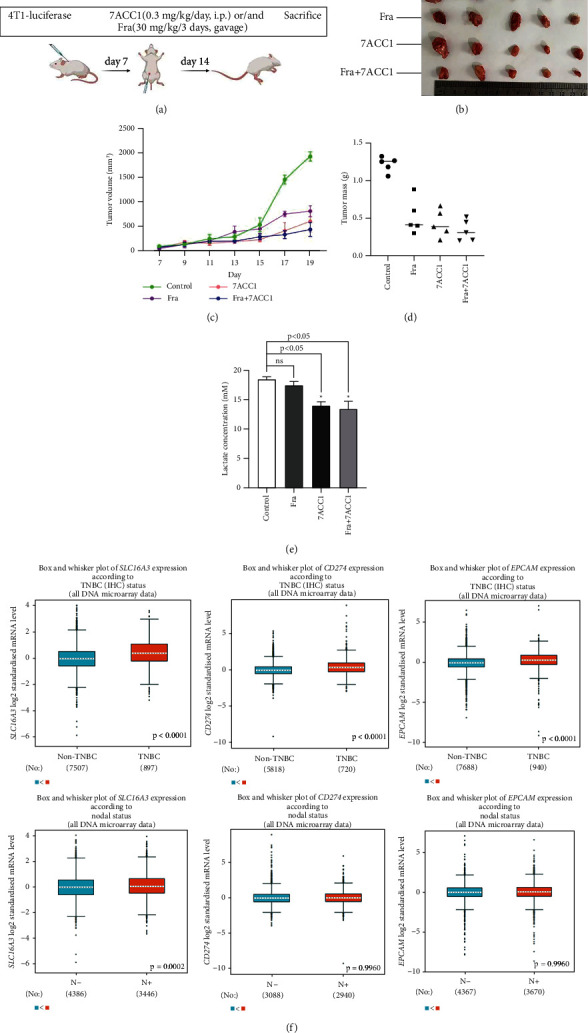
BALB/C mice inoculated with syngeneic 4T1 breast cancer cells and gene expression analysis of different breast cancer staging. (a) Syngeneic 4T1 breast cancer mice were established and 7ACC1 and/or fraxinellone were injected for 14 days. (b–e) Tumor volume and mass were measured, and corresponding lactate concentrations were tested. (f) bc-GenExMiner data present that MCT4^+^PD-L1^+^EpCAM^+^ axis expressed highly in TNBC status and nodal status.

To evaluate the effect of interdicting MCT4 or PD-L1 on in vivo tumor growth capacity, 7ACC1 and fraxinellone were used in BALB/C mice. There were four groups based on the pretreatment of the cells, which includes DMSO, 7ACC1, fraxinellone, and 7ACC1 combined with fraxinellone groups (Figure 6(a)). We found that the tumor volume and mass in the administration groups were smaller than the control group, and lactate concentration was lower than the control group (Figures 6(b)–6(e)). Furthermore, 7ACC1 combined with the fraxinellone group presented the largest effect on tumor inhibition.

In the last, we provided the occurrence of MCT4^+^PD-L1^+^EpCAM^+^ axis in breast cancer differentiation through bc-GenExMiner, revealing that high MCT4^+^PD-L1^+^EpCAM^+^ axis occurs in TNBC status and nodal status (Figure 6(f)), and the change was not obvious in ER-positive, PR-positive, and HER2-positive breast cancer (Figure S5).

## 4. Discussion

Glycolytic tumors including TNBC [40] express transporters such as MCT4 that facilitate the discharge of lactate to support metabolism, which contributes to the acidification of the local tumor environment [41]. Studies have shown that MCT4 is highly expressed in TNBC tumor stroma [28] and MCT4 expression in microenvironment estimates poor prognosis [42]. Lactate can stimulate macrophages turning toward the anti-inflammatorytumor-promoting M2 phenotype [34], which represents a potential correlation between the metabolic environment and immune environment. Heretofore, we found that MCT4/lactate could influence PD-L1 expression through GSK3*β*/*β*-catenin, but the molecule influenced directly by lactate stimulation was not identified. Whether lactate receptor GPR81 or transporter MCT1 is involved needs further exploration.

Glycosylation is a ubiquitous post-translated modification. PD-L1 contains 3 mainly N-glycosylation sites: N192, N200, and N219 [43]. It has been reported that STT3-dependent PD-L1 N-glycosylation could stabilize and upregulate PD-L1 via inducing EMT/*β*-catenin [44]. Previous studies also proved that glycosylation is easily affected by extracellular pH and promoted by acidic environment, which promotes evasion of T-cell immunity in turn [45]. Our study proved that PD-L1 was glycosylated and attributed by lactate stimulation in TBcls, which enhances the cellular viability. It is known that protein glycosylation processes mostly involve sequential concerted steps in the endoplasmic reticulum (ER) and the Golgi system (33087899), but the mechanism of how lactate/GSK3*β*/*β*-catenin promotes the process of PD-L1 glycosylation remains unknown. Meanwhile, the inhibition of PD-L1 glycosylation impairs the cellular viability, which may inhibit a mode of tumor cell death.

Our study reveals that the addition of lactate increases the expression and stability of PD-L1 and boosts the cell viability by inactivating GSK3*β* and releasing *β*-catenin into the nucleus. It reveals that the inhibition of the pathway can be a potential strategy to improve the therapeutic efficacy of ICB. Besides, our findings may contribute to further refinement of TNBC typing. In January 2021, Shao's team conducted a comprehensive analysis of TNBC multi-omics data (*n* = 465) [46]. Based on differences in metabolic pathways, the study classified TNBC into MPS1, MPS2, and MPS3 types: MPS1, the lipogenic subtype with upregulated lipid metabolism; MPS2, the glycolytic subtype with upregulated carbohydrate and nucleotide metabolism; and MPS3, the mixed subtype with partial pathway dysregulation. It also used in vivo experiments to confirm that the combination of glycolysis inhibitors and anti-PD-1 treatment was effective in antitumor treatment; for instance, anti-LDH treatment makes MPS2 TNBC more sensitive to immune checkpoint inhibitors. The expression of MCT4 and PD-L1 can be a biomarker to predict the specific subtype of TNBC so that the effective treatment can be applied.

## 5. Conclusions

We found that PD-L1 had a high sensitivity to MCT4 expression and lactate concentrations in TNBC, and lactate mainly promoted the N-glycosylation of PD-L1 by activating WNT pathway to incur its stable accumulation and expression on the cell membrane. Overall, studies targeting tumor metabolism and PD-L1 glycosylation may offer new strategies to combat TNBC and possibly other types of cancer.

## Figures and Tables

**Table 1:** Quantitative real-time PCR primer sequences.

Gene	Primer sequences
GAPDH (197bp)	5′-GGAGCGAGATCCCTCCAAAAT-3′ (forward)
	5′-GGCTGTTGTCATACTTCTCATGG-3′ (reverse)

MCT4 (225bp)	5′-CCATGCTCTACGGGACAGG-3′ (forward)
	5′-GCTTGCTGAAGTAGCGGTT-3′ (reverse)

PD-L1 (120bp)	5′-TGGCATTTGCTGAACGCATTT-3′ (forward)
	5′-TGCAGCCAGGTCTAATTGTTTT-3′ (reverse)

**Table 2:** MCT4 small-guide RNA (sgRNA) oligo-sequences.

Groups	sgRNA sequences
Negative control	5′-GAACGACTAGTTAGGCGTGTA-3′
MCT4 targeting sgRNA1	5′-TGTTACTATCGCAGCCCTGT-3′
MCT4 targeting sgRNA2	5′-GTAGGTCCCCCCGTGCACTG-3′

## Data Availability

The data and materials used in this study used to support the findings of this study are available from the corresponding author upon request.

## Abbreviations

TNBC: Triple-negative breast cancer
MCT4: Monocarboxylate transporter protein 4
ER: Estrogen receptor
PR: Progesterone receptor
HER2: Human epidermal growth factor receptor-2
BC: Breast cancer
PD-1: Programmed cell death 1
PD-L1: Programmed cell death 1 ligand 1
ICB: Immune checkpoint blockade
TBcs: TNBC cells
TBps: TNBC patients
CTLA-4: Cytotoxic T-lymphocyte-associated antigen-4
OCR: Oxygen consumption rate
mIHC: Multi-immunohistochemistry staining
PVDF: Polyvinylidene difluoride
WB: Western blot
qPCR: Quantitative real-time PCR.

## Supplementary Materials

Supplementary Materials
*Figure S1*. Two gene sets closely related to SLC16A3 (MCT4) or CD274 (PD-L1). *Figure S2.* Morphological relationship between MCT4 and PD-L1. *Figure S3 or Figure S4*. Expression of *SLC16A3* or *CD274* in human TNBC cell lines MDA-MB-231, MDA-MB-468, and BT-549. *Figure S5.* Expression of MCT4(SLC16A3)/PD-L1(CD274)/EPCAM in different subtypes of breast cancer. *Material S1*. mIHC dying protocol. *Material S2.* Analysis of tissue imaging.
