# Supplementary material for: MCT4/Lactate Promotes PD-L1 Glycosylation in Triple-Negative Breast Cancer Cells
Source: J Oncol. 2022 Sep 26;2022:3659714. doi: 10.1155/2022/3659714 (PMC9529401; doi:10.1155/2022/3659714)

**Supplementary Figures**

**MCT4/lactate promotes PD-L1 glycosylation in Triple-Negative Breast Cancer cells**

Xianxian Duan^1^, Yu Xie^1^, Jing Yu^1^, Xiao Hu^4^, Zhanzhao Liu^1^, Ning Li^5^, Junfang Qin^1^, Lan Lan^3^, Mengci Yuan^1^*, Zhanyu Pan^3^*, Yue Wang^1, 2^*

^1^School of Medicine, Nankai University, Tianjin 300071, China

^2^Tianjin Key Laboratory of Oral and Maxillofacial Function Reconstruction, Hospital of Stomatology, Nankai University, Tianjin 300041, China

^3^Tianjin Medical University, Tianjin Cancer Hospital, Tianjin 300060, China

^4^State Key Laboratory of Medicinal Chemical Biology & College of Pharmacy, Nankai University, Tianjin300350, China

^5^Institute of Disaster and Emergency Medicine, Tianjin University, Tianjin, 300072, China

*Co-corresponding authors: These authors contributed equally to this work. ymc7048@nankai.edu.cn (Mengci Yuan)[,](mailto:wangyue@nankai.edu.cn,) tjpanzhanyu@medmail.com.cn (Zhanyu Pan), wangyue@nankai.edu.cn (Yue Wang)

**Fig. S1** Two gene sets closely related to *SLC16A3* (MCT4) or *CD274* (PD-L1) were obtained from String online database. (A) an entity-relationship diagram was described, (B)a heat map of the enrichment analysis and (C) a merged enrichment analysis was described, respectively.

**A**


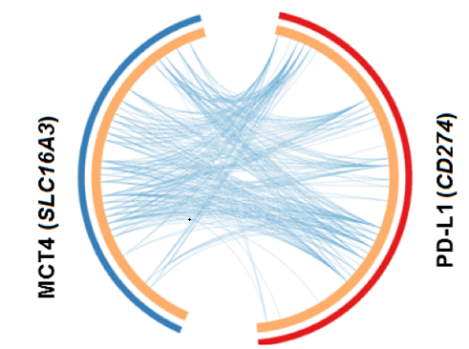


**B**


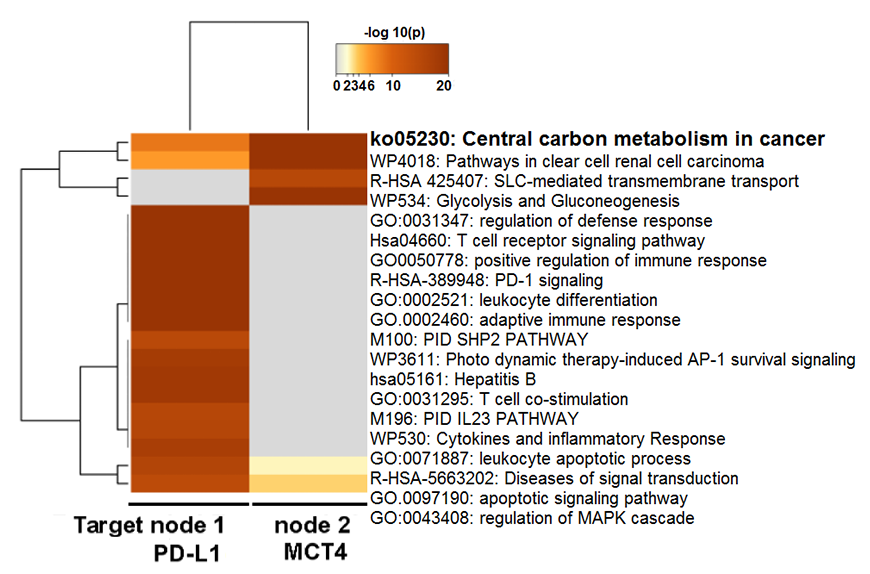


**C**


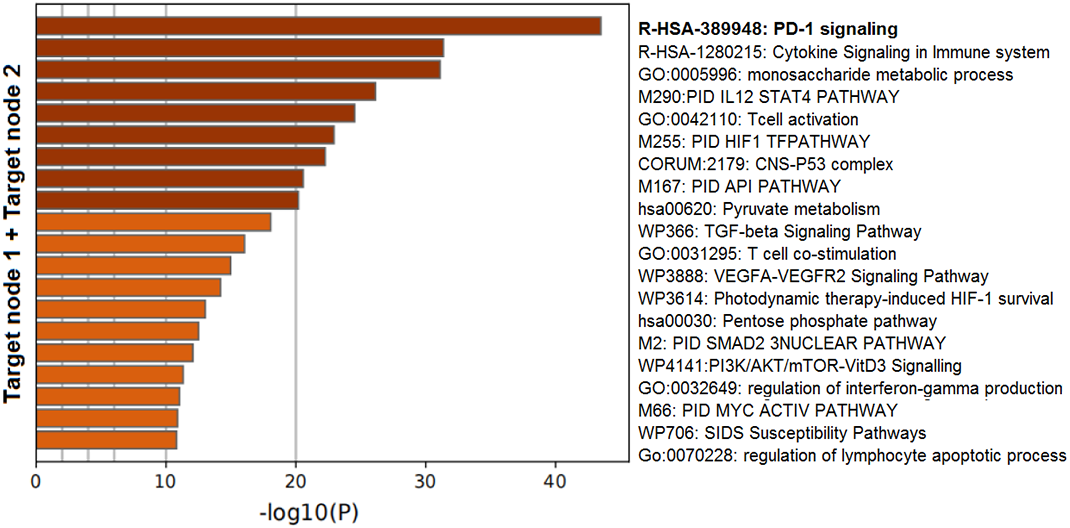


**Fig. S2** The morphological relation between MCT4 and PD-L1 was investigated using the consecutive method for demonstrating mIHC on the same section in a TNBC patients’ tumor tissue (case 2) was quantified.


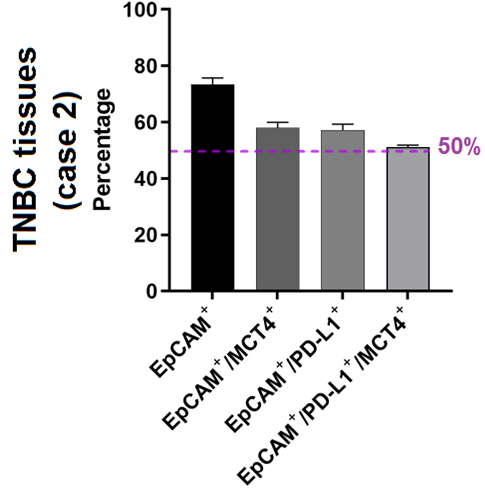


**Fig. S3** Western blot (WB) exhibited the *SLC16A3* gene could express in cells, the expressed MCT4 protein possess specificity in human TNBC cell lines MDA-MB-231, MDA-MB-468 and BT-549.


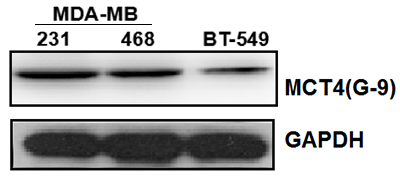


**Fig. S4** Western blot(WB) exhibited the *CD274* gene could express in  cells, the expressed PD-L1 protein possess specificity in human TNBC cell lines MDA-MB-231, MDA-MB-468 and BT-549.


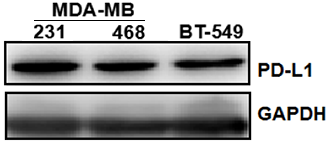


**Fig S5.** Expression of MCT4(SLC16A3) in different subtypes of breast cancer


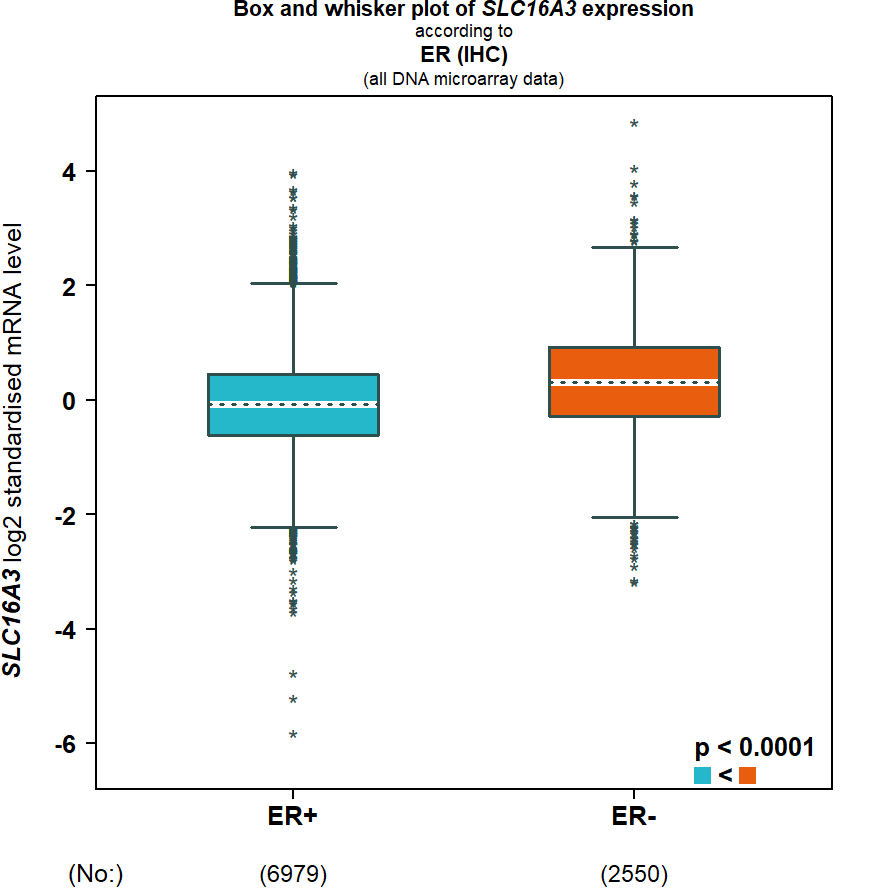

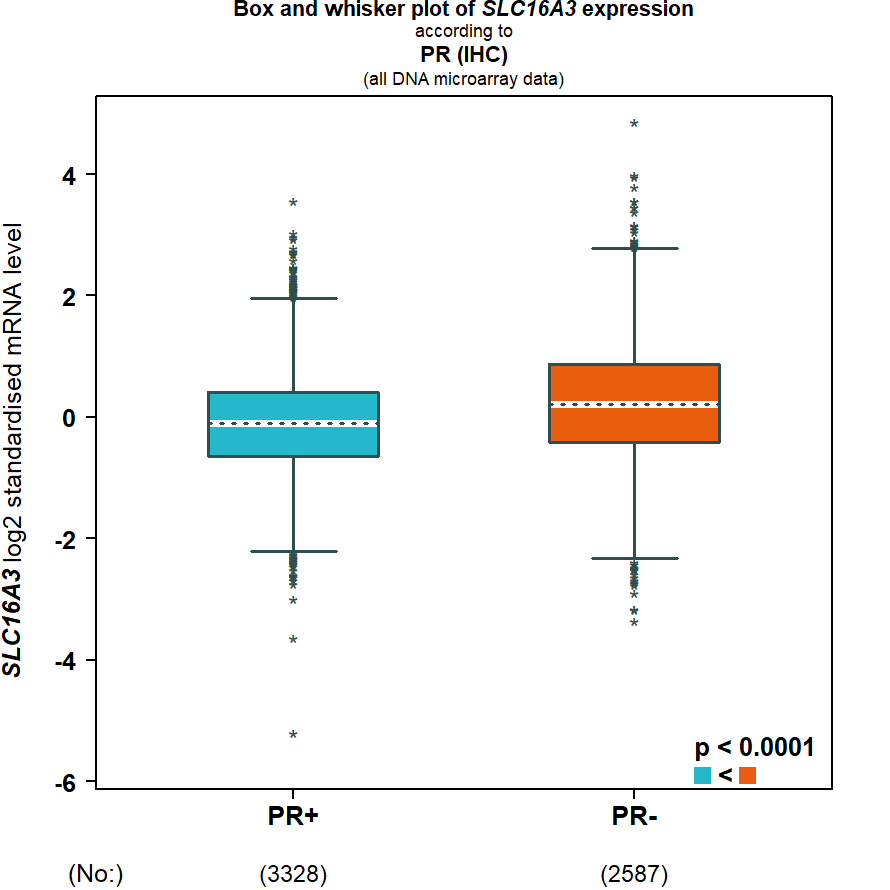


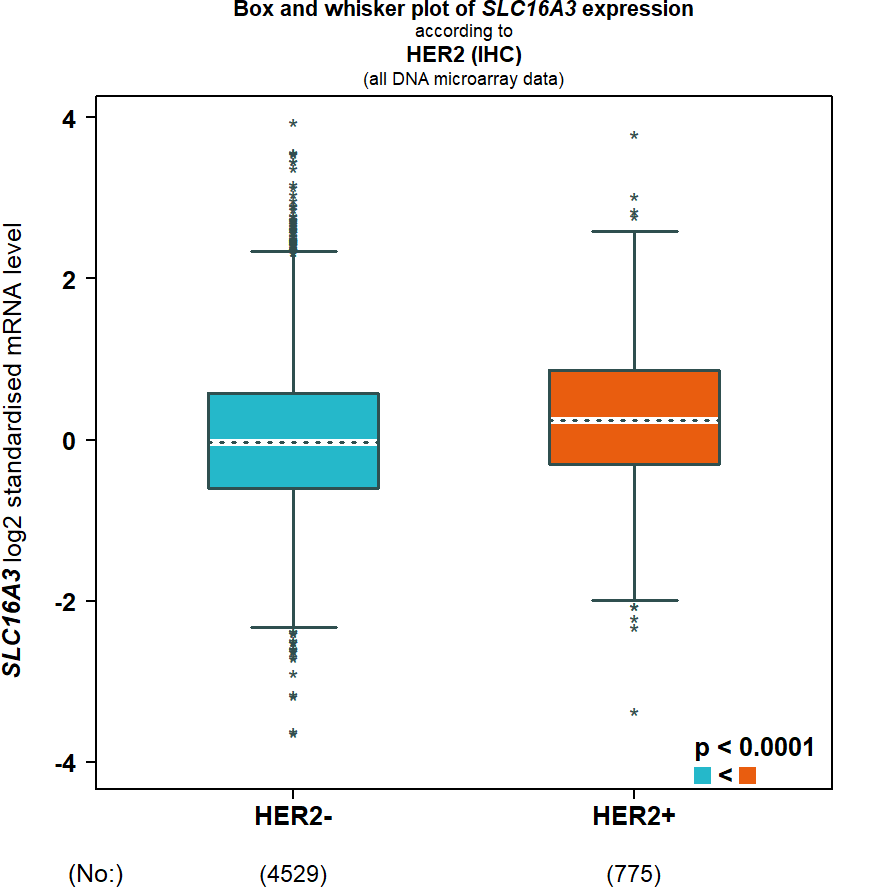


**Fig S5.** Expression of PD-L1(CD274) in different subtypes of breast cancer


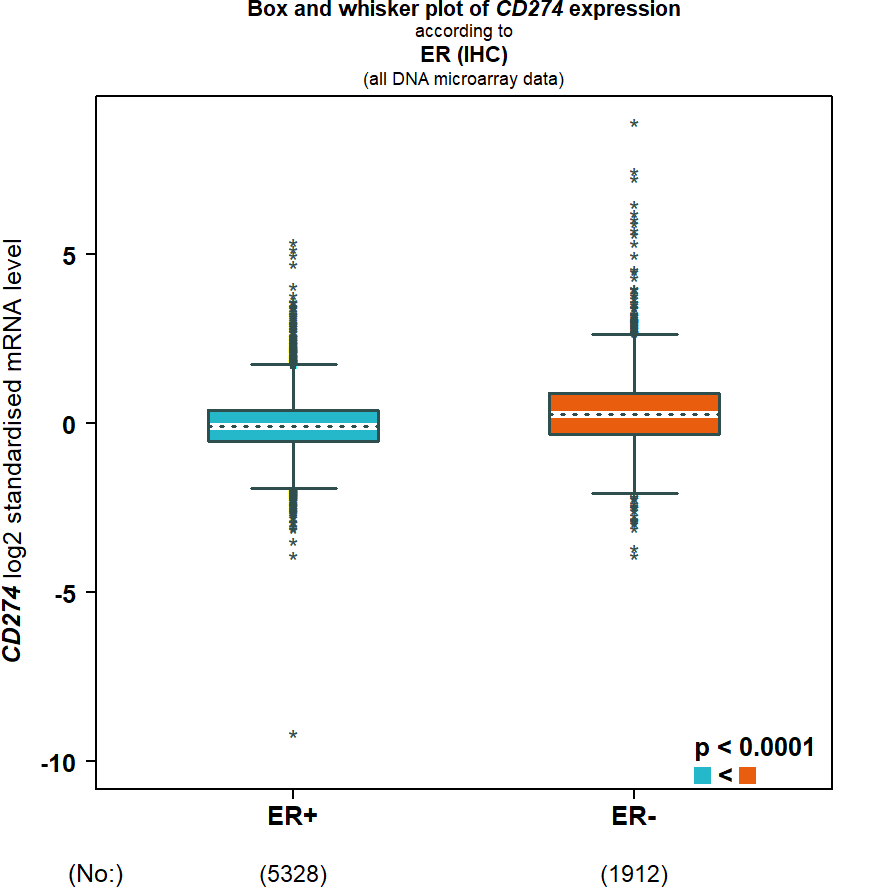


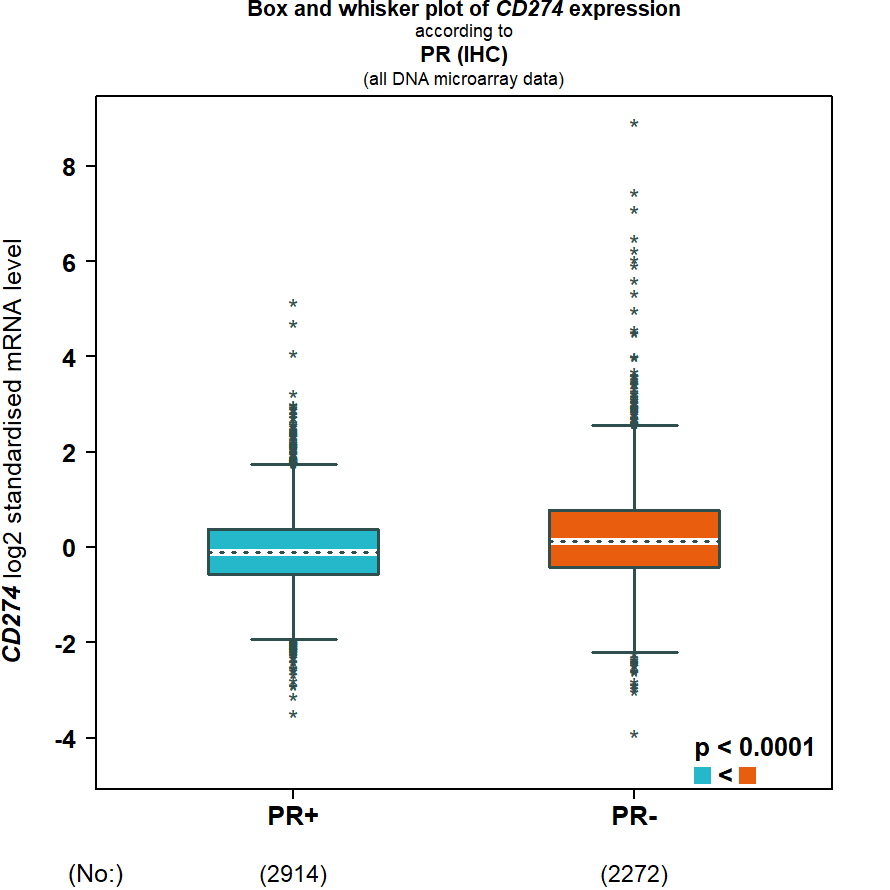


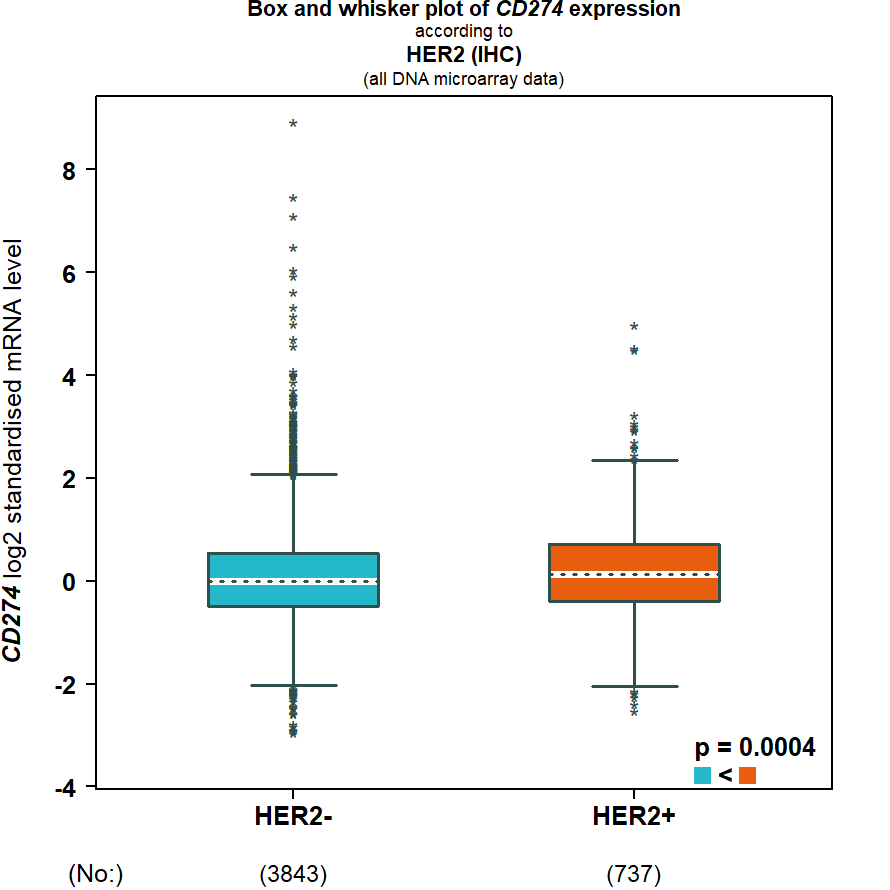


**Fig S5.** Expression of EPCAM in different subtypes of breast cancer


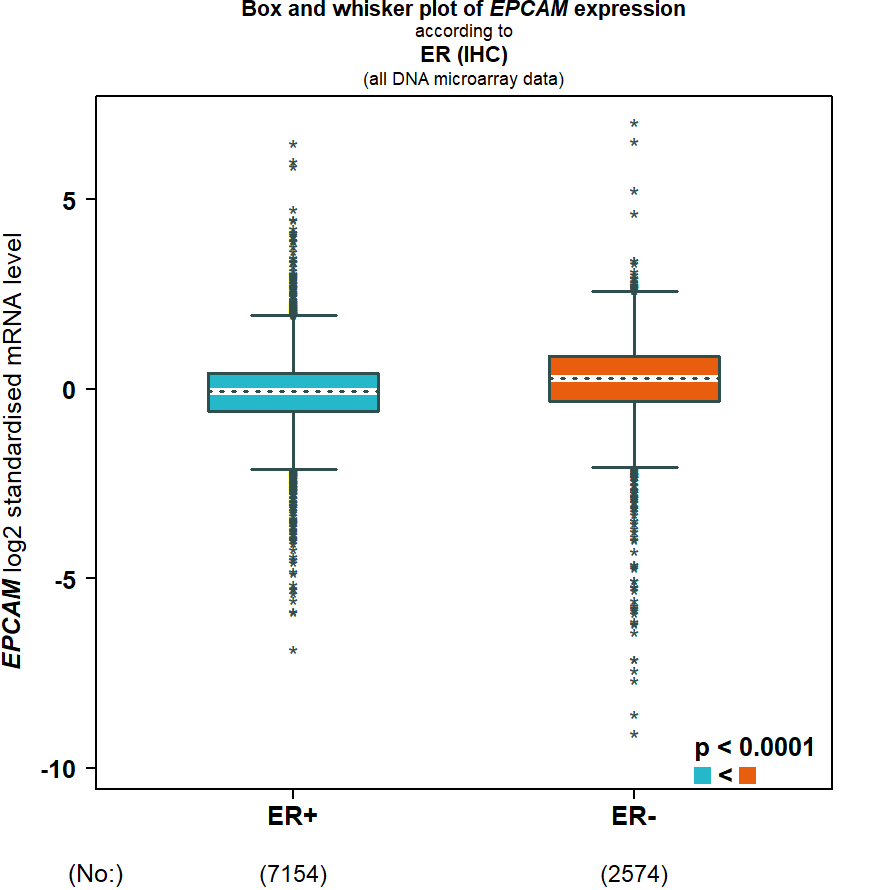


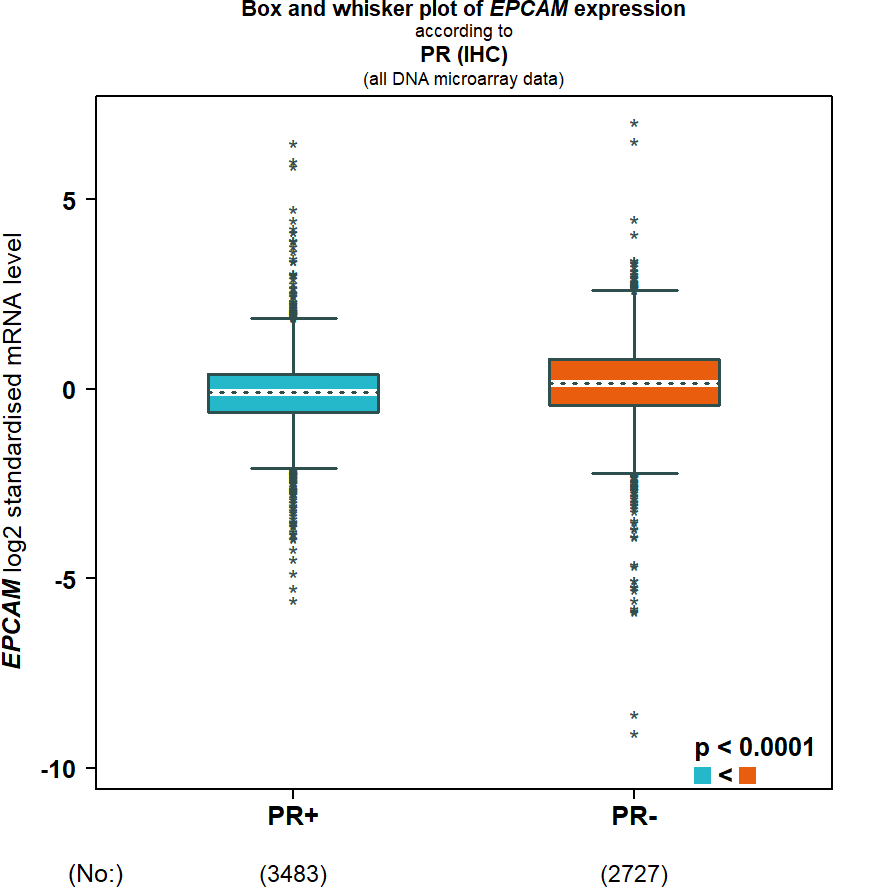


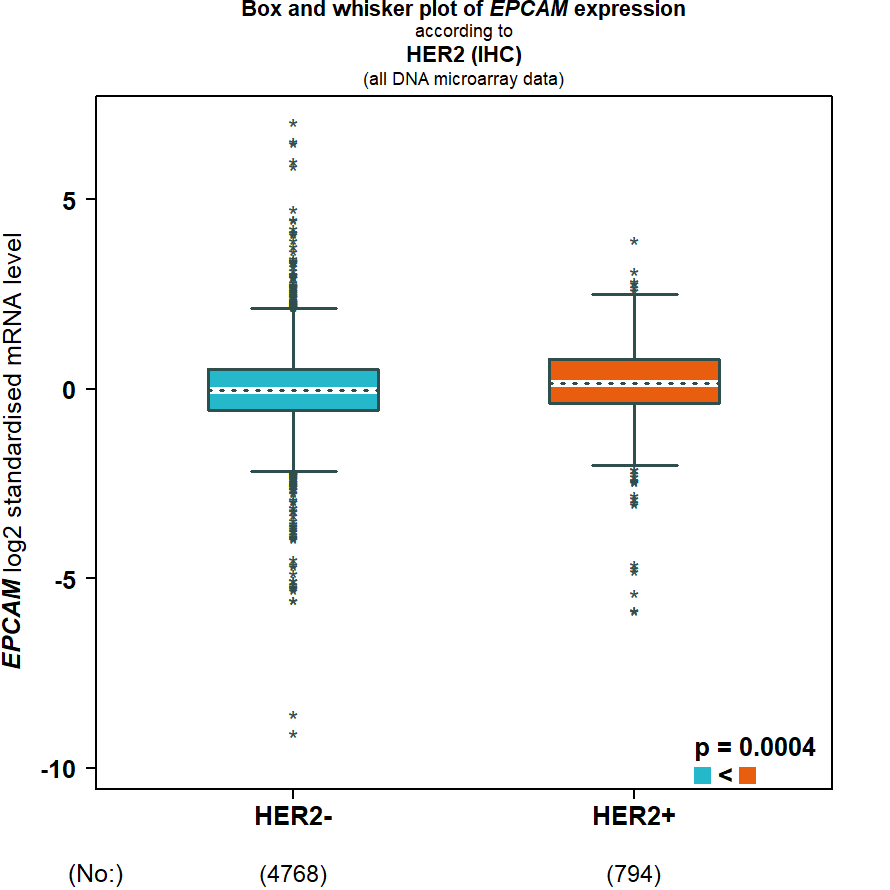

Supplement: Supplementary Materials — Figure S1. Two gene sets closely related to SLC16A3 (MCT4) or CD274 (PD-L1). Figure S2. Morphological relationship between MCT4 and PD-L1. Figure S3 or Figure S4. Expression of SLC16A3 or CD274 in human TNBC cell lines MDA-MB-231, MDA-MB-468, and BT-549. Figure S5. Expression of MCT4(SLC16A3)/PD-L1(CD274)/EPCAM in different subtypes of breast cancer. Material S1. mIHC dying protocol. Material S2. Analysis of tissue imaging. [file 3659714.f1.zip › Supplementary Figures S1-S5.docx]
