# Supplementary material for: MCT4/Lactate Promotes PD-L1 Glycosylation in Triple-Negative Breast Cancer Cells
Source: J Oncol. 2022 Sep 26;2022:3659714. doi: 10.1155/2022/3659714 (PMC9529401; doi:10.1155/2022/3659714)
Supplement: Supplementary Materials — Figure S1. Two gene sets closely related to SLC16A3 (MCT4) or CD274 (PD-L1). Figure S2. Morphological relationship between MCT4 and PD-L1. Figure S3 or Figure S4. Expression of SLC16A3 or CD274 in human TNBC cell lines MDA-MB-231, MDA-MB-468, and BT-549. Figure S5. Expression of MCT4(SLC16A3)/PD-L1(CD274)/EPCAM in different subtypes of breast cancer. Material S1. mIHC dying protocol. Material S2. Analysis of tissue imaging. [file 3659714.f1.zip › Supplementary Material S1.pdf]

WiSee Biotechnology

WiSee Biotechnology Co., Ltd

Customer Sample Analysis

1. Project title:

mIHC detection of indicating molecules in tumor tissues

2. Date:

May 2nd 2021

3. mIHC dying protocal:

SN	Antigen Retrieval	Primary Antigen	Dilution Ratio & times	Secondary Antigen	Dilution Ratio & times	Dyes	Dilution Ratio & times	Termi-nation
1	AR9	MCT4 (G-7) or (G-9)	1:100 & 4℃ at overnight	Goat anti-mouse IgG (H+L)	1:100 & 10min	Neon TSA520	1:100 & 10min	Stop solution
2	AR9	PD-L1	1:400 & 1h	Goat anti-rabbit IgG (H+L)	1:100 & 10min	Neon TSA620	1:100 & 10min	Stop solution
3	AR9	EpCAM (D9S3P)	1:400 & 1h	Goat anti-rabbit IgG (H+L)	1:100 & 10min	Neon TSA570	1:100 & 10min	Stop solution
