# Supplementary material for: MCT4/Lactate Promotes PD-L1 Glycosylation in Triple-Negative Breast Cancer Cells
Source: J Oncol. 2022 Sep 26;2022:3659714. doi: 10.1155/2022/3659714 (PMC9529401; doi:10.1155/2022/3659714)
Supplement: Supplementary Materials — Figure S1. Two gene sets closely related to SLC16A3 (MCT4) or CD274 (PD-L1). Figure S2. Morphological relationship between MCT4 and PD-L1. Figure S3 or Figure S4. Expression of SLC16A3 or CD274 in human TNBC cell lines MDA-MB-231, MDA-MB-468, and BT-549. Figure S5. Expression of MCT4(SLC16A3)/PD-L1(CD274)/EPCAM in different subtypes of breast cancer. Material S1. mIHC dying protocol. Material S2. Analysis of tissue imaging. [file 3659714.f1.zip › Supplementary Material S2.pdf]

# Analysis of tissue imaging

| Image tab | Total cells | $MCT4^+$ cells | $EpCAM^+$ cells | $PDL1^+$ cells | $MCT4^+/EpCAM^+/PDL1^+$ cells | $MCT4^+$ Positive Cells % | $EpCAM^+$ Positive Cells % | $PDL1^+$ Positive Cells % | $MCT4^+/EpCAM^+/PDL1^+$ Positive Cells % |
|---|---|---|---|---|---|---|---|---|---|
| MCT4 (G-9) | 720462 | 402290 | 475504 | 396405 | 366715 | 55.84 | 66 | 55.02 | 50.9 |
| PD-L1 EPCAM | 719867 | 418022 | 473672 | 410663 | 374330 | 58.07 | 65.8 | 57.05 | 52 |
| | 732514 | 438902 | 476134 | 434456 | 374021 | 59.92 | 65 | 59.31 | 51.06 |
| MCT4 (G-7) | 1103817 | 602242 | 835258 | 588138 | 560407 | 54.56 | 75.67 | 53.28 | 50.77 |
| PD-L1 EPCAM | 1094891 | 602190 | 803759 | 581572 | 553795 | 55.00 | 73.41 | 53.12 | 50.58 |
| | 1138465 | 646534 | 809562 | 570371 | 592001 | 56.79 | 71.11 | 50.10 | 52.03 |

WiSee Biotechnology

WiSee Biotechnology Co.,Ltd
